# Fabrication of PMMA-PS Fiber Films with Superhydrophobic Properties Assisted by Ultrasonic and Magnetic Field Coupling Electrospinning

**DOI:** 10.3390/polym18091075

**Published:** 2026-04-29

**Authors:** Hao Yin, Shiyao Wang, Jingbin Liu, Xiao Wu, Yue Hou, Wenwen Zhang, Dan Peng

**Affiliations:** 1 Naval University of Engineering, Wuhan 430033, China; 1508062001@nue.edu.cn (H.Y.); 18163307200@163.com (S.W.); 2320231027@nue.edu.cn (J.L.); penny8866@163.com (Y.H.); zwwada@163.com (W.Z.); 2 School of Mechanical Engineering and Automation, Wuhan Textile University, Wuhan 430200, China; wuxiao@wtu.edu.cn

**Keywords:** electrospinning, PMMA/PS, fiber films, spindle, superhydrophobic

## Abstract

Superhydrophobic fiber films, as a typical superhydrophobic material, have advantages such as self-cleaning, non-wettability, and pollution resistance. They can be widely used in oil-water separation, antibacterial, anti-pollution, anti-icing, and self-cleaning fields. Traditional electrospun superhydrophobic fiber films face difficulties in fabricating fibers with large contact angles due to the non-Newtonian fluid flow and Taylor cone jet trajectory limitations. To address this challenge, this study develops a novel ultrasonic-magnetic field coupling electrospinning strategy for fabricating poly(methyl methacrylate)-polystyrene (PMMA-PS) fibrous films with enhanced superhydrophobicity. Physical, chemical, and contact angle measurements were used to analyze the morphology, composition, and hydrophobic properties of the fabricated films. The results showed that by controlling the blend ratio of PMMA and PS and optimizing the electrospinning process with ultrasonic vibration and magnetic field coupling, PMMA-PS fibers with better fiber refinement, closer spindle-shaped arrangements, and significantly increased roughness were successfully fabricated. When using 15% PMMA and 15% PS solutions, the static contact angle of the resulting fiber films reached 173.1°, demonstrating the best superhydrophobicity. The study suggests that optimizing the surface morphology of the nanofibers is an effective method to improve hydrophobicity and provides a new approach for fabricating superhydrophobic fiber films.

## 1. Introduction

Poly(methyl methacrylate) (PMMA) and polystyrene (PS) are both polymers characterized by distinct physical and chemical properties. PMMA is recognized for its high optical transparency and exceptional mechanical properties, rendering it suitable for a wide range of applications in optical and medical fields [1,2]. In contrast, PS exhibits notable attributes such as non-toxicity, biocompatibility, and chemical inertness [3]. The combination of these polymers offers a synergistic potential for diverse applications, including medical applications (e.g., bone bonding and dental applications) and industrial sectors such as automotive manufacturing [4,5]. Research has demonstrated that PMMA/PS blends exhibit enhanced mechanical properties, including improved tensile strength and conductivity [6]. Furthermore, PMMA-PS fiber films, characterized by superior optical properties [7,8], mechanical stability [9], and chemical resistance [10], represent promising functional materials for advanced applications, and chemical resistance, represent promising functional materials for advanced applications. These materials hold significant potential for widespread use in advanced materials science and engineering.

Recently, superhydrophobic surfaces have attracted substantial interest due to their potential applications in self-cleaning surfaces, microfluidics, and special waterproof fabrics that allow sweating. It is well-established that superhydrophobicity critically depends on the combination of micro- and nanostructures on the surface. The key factors in the surface synthesis process are the parameters that define the surface conformation. As a new material, PMMA-PS fiber films are believed to possess potential superhydrophobic properties [11]. Therefore, how to achieve superhydrophobicity while maintaining their other properties is one of the primary research focuses in the fabrication of PMMA-PS fiber films. Superhydrophobic films are typically made from hydrophobic materials. PMMA is considered a hydrophilic polymer (with an inherent contact angle below 90°), and its water contact angle (CA) is 68°. However, Ma et al. [12] achieved a superhydrophobic PMMA film with a contact angle of 154° by treating a PMMA/PS mixed film with a warm selective solvent, cyclohexane. The transition from hydrophilic to superhydrophobic surfaces was found to be due to the synergistic effect of the micro-nano structures and the reorientation of the side groups of PMMA chains at the top.

To achieve superhydrophobicity on the PMMA-PS material surface, traditional methods involve direct surface modification. Common methods include both physical and chemical approaches. Physical methods include spin-coating blend films [13,14], plasma surface treatment [13], surface etching [14], and nanoparticle deposition to modify the microstructural surface morphology and achieve superhydrophobic effects. Chemical methods include phase separation and selective solvent treatment methods [12,15], surface coatings [16], chemical modification [17], and adding nanoparticles by spin-coating [10,18] and dual-spray techniques [19] to alter the chemical properties of the surface. Additionally, self-assembly techniques can form specific micro-nano structures on PMMA-PS surfaces, leading to superhydrophobicity [20]. Common self-assembly techniques include solution immersion, solvent evaporation, and templating methods. However, these traditional methods face numerous issues, such as long preparation cycles, high costs, complex processes, uneven fiber structures, and unstable film quality. Finding an efficient, low-cost, and simple fabrication method has become a key challenge to be addressed.

Electrospinning, as a fiber preparation technique, is one of the simplest, most convenient, efficient, and cost-effective methods available, particularly in the production of fibers with high axial strength and large specific surface area [21]. Moreover, electrospun fiber films exhibit several advantages, including reproducibility, uniform fiber alignment, water capture ability, high “rose petal effect,” significant adhesion, and good hydrophobicity, making them a suitable candidate for liquid droplet directional transfer films [22,23]. Nevertheless, as the PMMA/PS electrospun fiber film technology continues to mature, two critical technical challenges need to be addressed: how to effectively regulate the non-Newtonian fluid flow within the spinning solution [24], and how to precisely control the movement trajectory of the Taylor cone and jet during the fabrication process [22,25]. The spinnability of high-concentration spinning solutions and the associated performance have been a subject of extensive research. As the concentration or viscosity of the spinning solution increases, the strength and mechanical properties of the spun fibers also increase, and the production efficiency improves significantly [26]. However, high-concentration spinning solutions not only clog the channels but also cause a drastic reduction in the flowability during the electrospinning process. The higher the viscosity of the spinning solution, the poorer its flowability, and the higher the required electric field force [22]. Therefore, achieving a decrease in the viscosity of the spinning solution while improving its flowability and production efficiency to obtain excellent performance is currently one of the hotspots in the electrospinning process for fiber film fabrication.

Ultrasonic vibration, as an effective solution, can address the issue of viscosity reduction in high-concentration spinning solutions. During ultrasonic treatment, cavitation effects (the rapid formation, growth, and collapse of unstable bubbles in the liquid) generate significant energy, breaking large aggregates of nanoparticles (NPs) into smaller dispersed particles and increasing the specific surface area of the nanoparticles, thus enhancing their effectiveness [27]. Baig et al. found that TiO_2_ NPs can be well-dispersed and uniformly attached to graphene oxide (GO) sheets under ultrasonic conditions. This method increases the interaction between NPs and GO sheets, producing a larger surface area and further promoting adsorption and photocatalysis [28]. Cao et al. [29] discovered that ultrasonic-assisted electrospinning of polyacrylonitrile (PAN) fiber films effectively reduced the fiber diameter, significantly improved the spinnability of the PAN solution, and optimized the surface morphology and mechanical properties of the PAN fiber films. Si et al. [30] treated high-viscosity polyvinyl alcohol (PVA) spinning solutions with ultrasonic vibration and found that this process reduced the fiber diameter, and the viscosity and conductivity of the PVA solution were strongly correlated with the ultrasonic vibration time and intensity. Qiang et al. [31] observed that the dynamic rheological properties, conductivity, surface tension, and Taylor cone shape of PAN spinning solutions were closely related to the duration of ultrasonic vibration. These studies show that ultrasonic treatment can significantly reduce the viscosity of the spinning solution and greatly improve its spinnability.

This study addresses the challenges associated with electrospinning PMMA-PS fiber membranes with superhydrophobic properties, particularly the issue of improving the surface structure to enhance hydrophobicity. We aim to develop a cost-effective and efficient electrospinning process using ultrasonic vibration and magnetic field coupling to enhance the fiber structure and surface roughness. This innovative approach is expected to produce PMMA-PS fiber membranes with significantly improved superhydrophobic properties, which have vast potential for applications in self-cleaning surfaces, waterproof coatings, and microfluidics. By optimizing the electrospinning parameters and introducing auxiliary mechanisms like ultrasonic vibration and magnetic fields, we seek to overcome the limitations of traditional methods and offer a new solution to produce high-performance, superhydrophobic fiber membranes.

## 2. Experimental Section

Materials. The PMMA used was of analytical grade with a molecular weight of 200,000, sourced from Shanghai Aladdin Biochemical Technology Co., Ltd. Shanghai, China. Similarly, PS, also of analytical grade, had a molecular weight of 80,000 and a particle size ranging from 7 to 40 nm. Fumed silica (SiO_2_, amorphous, with a primary particle size of 10–20 nm, specific surface area of ~200 m^2^/g). The solvent DMF (N,N-Dimethylformamide) was obtained from the same supplier. Additionally, Polyacrylonitrile (PAN), with a molecular weight of 250,000, was incorporated in some preparations.

Preparation of fibrous PMMA membranes. To prepare the PMMA precursor solution, 25% (*w*/*v*) PMMA was dissolved in 30 mL of DMF and stirred for 12 h to ensure complete dissolution. The solution was then transferred to the A-pump of the electrospinning apparatus.

Electrospinning parameters. A pump speed of 0.3 mm/s, a needle-to-collector distance of 15 cm, and an applied voltage of 15 kV. The electrospinning process lasted for 180 min. After electrospinning, the film was vacuum-dried for 8 h to obtain the PMMA film. PMMA was also synthesized by mixing 20 mL of MMA monomer with 20 mg of benzoyl peroxide (BPO) and 160 mg of p-toluidine. The mixture was heated to 90 °C, and polymerization was initiated after 20–30 min. The resulting PMMA was isolated or used in its viscous form for further reactions. For PS preparation, 10 mL of styrene monomer was mixed with 10 mg of BPO and 160 mg of p-toluidine. The mixture was heated to 90 °C, and polymerization started after 30 min. The resulting PS was isolated or used for further reactions.

To optimize the surface for maximal hydrophobicity, a coaxial electrospinning configuration was chosen in preference to a simpler single-nozzle blending technique. The coaxial electrospinning method facilitates the production of core–shell fiber architectures, enabling precise localization of hydrophobic polystyrene (PS) and silica (SiO_2_) components within the outer sheath layer. This strategy ensures a consistently hydrophobic surface by preventing the migration of the more hydrophilic poly(methyl methacrylate) (PMMA) to the fiber exterior, a phenomenon that may occur in blended solutions. Furthermore, the unique fluid dynamics inherent to the coaxial jet are instrumental in forming the spindle-knot morphology, which is critical for establishing the hierarchical surface roughness necessary to achieve superhydrophobic properties [1].

Preparation of fibrous PMMA/PS membranes. To prepare the PMMA/PS blend, 0.5 mL of PMMA (65 mg) and 0.5 mL of PS (65 mg) were mixed with acetone and sonicated for 20 min at room temperature. The PMMA solution was prepared by dissolving PMMA beads in acetone. The mixture was gently heated to 50 °C in a sealed container under magnetic stirring for 4 h to accelerate the dissolution process. The PS solution was prepared by dissolving 15 wt% polystyrene beads in a binary solvent mixture of N,N-dimethylformamide (DMF) and tetrahydrofuran (THF) (7:3 *v*/*v*) under magnetic stirring at 40 °C for 6 h to ensure complete dissolution. The resulting PMMA/PS foam polymer was heated at 75 °C with stirring for 10 min to form a viscous liquid, which was used in the subsequent steps. Solution A contains 15% PMMA, and Solution B contains 12% PS. Solution B was stirred for 8 h, and then 0.5% SiO_2_ (vapor-phase type) was added and stirred for another 12 h to ensure full dissolution. Both solutions were placed in the A and B pumps of the electrospinning apparatus. The electrospinning parameters were set as follows: A-pump speed of 0.3 mm/s, B-pump speed of 0.5 mm/s, needle-to-collector distance of 15 cm, and voltage of 15 kV. Electrospinning was performed for 60 min. After electrospinning, the film was vacuum-dried for 8 h to obtain the final PMMA-PS fiber film. The PMMA-PS films were fabricated using different mass ratios of PMMA and PS. Various combinations of PMMA and PS concentrations were tested, including 5%, 10%, 15%, 20%, 25%, and 30% (*w*/*v*) for both PMMA and PS. In addition, different ultrasonic vibration and magnetic field conditions were applied during the electrospinning process to examine their effects on the fiber morphology and superhydrophobic properties. These parameters included ultrasonic vibration frequencies of 20 kHz to 30 kHz, with power levels of 1000 W and 1500 W, and magnetic field strengths of 0.2 T and 0.5 T. These parameters were systematically varied in preliminary experiments to optimize fiber morphology. The conditions reported in the subsequent sections (ultrasonic treatment at 21 kHz, 1000 W and a magnetic field of 0.2 T) were identified as optimal, providing the best balance between the formation of uniform spindle-knot structures, process stability, and the resulting superhydrophobic performance. Higher ultrasonic power (1500 W) led to undesirable solution heating, while a stronger magnetic field (0.5 T) offered only marginal improvements in fiber alignment at the cost of significantly increased experimental complexity.

Ultrasonic Vibration and Magnetic Field Coupling. The process for preparing PMMA-PS fiber films with ultrasonic vibration and magnetic field coupling is illustrated schematically. Unlike conventional electrospinning, the PMMA solution (Solution A) and PS solution (Solution B) were placed separately into sealed containers of the ultrasonic generator. Prior to electrospinning, the final polymer solution was subjected to a final ultrasonic treatment in a bath sonicator operating at 21 kHz for 30 min to eliminate any trapped air bubbles and ensure homogeneity. The solution was then immediately loaded into a 10 mL syringe for the electrospinning process. The entire process from sonication to the start of spinning was completed within 15 min to minimize solution relaxation. The ultrasonic bath was maintained at 25 ± 1 °C using a circulating water chiller. The temperature of the polymer solution after the 30 min sonication was measured to be 28 ± 2 °C, ensuring that viscosity changes due to significant heating were minimal. The solution was allowed to return to ambient temperature (25 °C) for 10 min before being loaded into the syringe pump. Each solution was then subjected to ultrasonic treatment at a frequency of 21 kHz, a power of 1000 W, and a duration of 2 h to reduce the viscosity and improve the mixture. After ultrasonic treatment, Solution A was placed in the A-pump, and Solution B was placed in the B-pump. During the electrospinning process, a magnetic field coupling device was added to ensure that the magnetic field remained active, which influenced the fiber morphology. After electrospinning, the fiber films were vacuum-dried for 8 h to yield the PMMA-PS fiber films.

Characterization. The surface morphology and structural characteristics of the fabricated fiber films were analyzed using Scanning Electron Microscopy (SEM) (FE-SEM scanning electron microscope, JSM-5600LV, JEOL, Tokyo, Japan). The diameter of the fibers was measured using Digital Micrograph software (DigitalMicrograph 3.0 (Gatan Inc., Pleasanton, CA, USA)). The elemental composition of the films was determined by Energy Dispersive X-ray Spectroscopy (EDS) (INCA X-MAX, Oxford Instruments, Abingdon, UK). The chemical structure of the films was examined by Fourier-transform infrared spectroscopy (FTIR) (FTIR-4100, Tokyo, Japan) with a scanning rate of 2 cm^−1^, 32 scans, and a testing range of 600 to 3000 cm^−1^. The contact angle (CA) of the electrospun films was measured using an automatic contact angle measurement instrument (CA200, Guangdong Beidou Instruments, Dongguan, China), with droplets of 1 µL, 3 µL, and 5 µL of deionized water to study the surface hydrophobicity. The water sliding angle (SA) was also measured by tilting the sample stage until the water droplet (10 μL) began to roll off. Water Contact Angle (WCA) measurements were performed using a sessile drop method on a goniometer (OCA 15EC, DataPhysics Instruments, Filderstadt, Germany) at ambient temperature (25 °C). A 5 µL droplet of deionized water was carefully deposited onto the membrane surface. To ensure a flat and stable measurement surface, the flexible membranes were mounted onto standard glass microscope slides using double-sided tape at the edges, avoiding any wrinkles or bumps in the measurement area. For each sample type, the WCA was measured at a minimum of 10 different locations to ensure statistical relevance. The reported values are the average ± standard deviation of these measurements. The X-ray diffraction (XRD) (Philips-Holland, PW 1729, Philips, Almelo, The Netherlands) was employed to study the crystalline and surface chemical properties of the prepared films.

## 3. Results and Discussion

### 3.1. Fabrication Strategy and Surface Morphology

**Figure 1:**
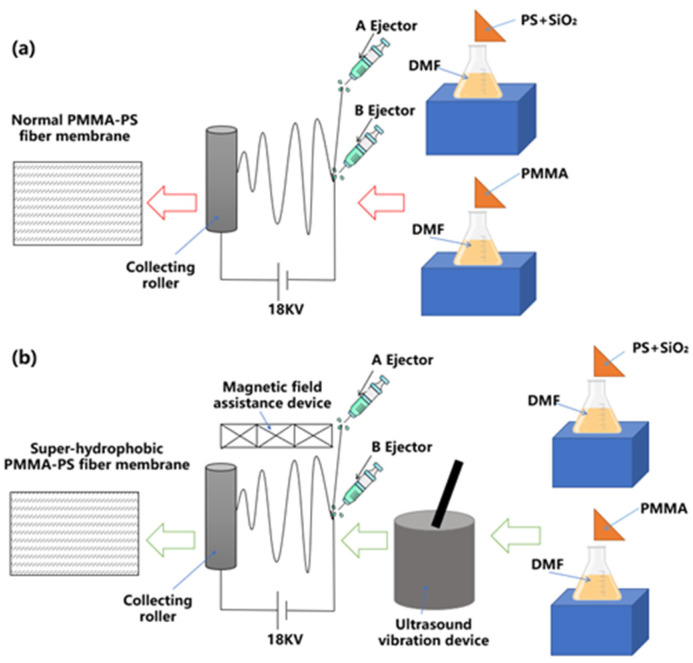
Schematic illustration of the two electrospinning routes used to fabricate PMMA-PS fibrous membranes. (**a**) Conventional coaxial electrospinning of PMMA (core) and PS/SiO_2_ (sheath) solutions in DMF is delivered through two ejectors under an 18 kV electric field toward a rotating collector. (**b**) Ultrasound and magnetic-field-coupled coaxial electrospinning: prior ultrasonic treatment (21 kHz, 1000 W) lowers solution viscosity, while an in situ magnetic-field assistance device stabilizes the charged jet and orients polymer chains. The synergy of the two auxiliary fields may produce spindle-decorated fibers with dual-scale roughness, resulting in a super-hydrophobic PMMA-PS membrane.

Figure 1 illustrates the preparation process of PMMA-PS fiber membranes via conventional electrospinning (Figure 1a) and the proposed ultrasonic vibration and magnetic field coupling-assisted electrospinning method (Figure 1b). In the conventional electrospinning setup, PMMA and PS precursor solutions are separately electrospun from two ejectors (A and B) onto a rotating collector, forming normal PMMA-PS fiber membranes. In contrast, the advanced method (Figure 1b) incorporates an ultrasonic vibration device to lower the viscosity of the spinning solutions and a magnetic field assistance device to improve molecular alignment and fiber deposition uniformity, resulting in superhydrophobic PMMA-PS fiber membranes. The integration of ultrasonic and magnetic field assistance into coaxial electrospinning reflects a rational, multi-scale engineering strategy to overcome conventional limitations of fiber uniformity, nanofiller distribution, and surface roughness control. This synergistic field-assisted approach may enable the direct, one-step fabrication of PMMA-PS membranes with exceptional superhydrophobic performance and well-controlled surface topographies, offering a promising route for the scalable production of high-performance functional materials for anti-wetting, self-cleaning, or breathable water-resistant textiles.

**Figure 2:**
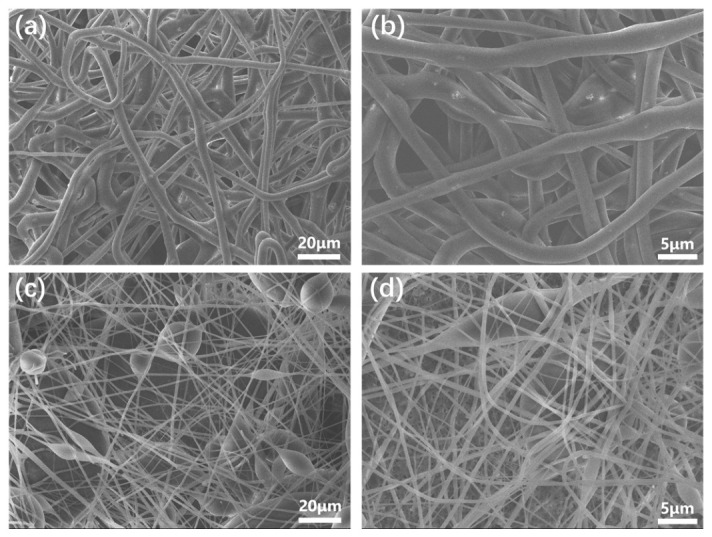
SEM micrographs highlighting the influence of the auxiliary fields on fibre morphology. (**a**,**b**) Conventional coaxial electrospinning yields randomly entangled PMMA-PS fibres with smooth surfaces and a narrow diameter distribution (average ≈ 3.3 µm); no spindle-bead defects are observed. (**c**,**d**) Coupling ultrasound (21 kHz, 1000 W) with a 0.2 T magnetic field produces a highly ordered network of finer fibres (thin segment ≈ 3.2 µm) decorated with densely packed fusiform spindle nodes (diameter ≈ 0.4 µm), endowing the mat with dual-scale roughness. Scale bars: 20 µm in (**a**,**c**) and 5 µm in (**b**,**d**).

Figure 2a,b show the morphology of the PMMA-PS fibrous membrane prepared by conventional electrospinning. The fibers are randomly oriented and densely interwoven, with a relatively uniform diameter distribution. As seen in the higher-magnification image (Figure 2b), the fiber surfaces are smooth and free of beaded structures, and the fiber diameters are fairly consistent (average diameter ~3.327 ± 0.26 μm). In contrast, the fibrous membrane produced under the coupled ultrasonic vibration and magnetic field (Figure 2c,d) exhibits a markedly different morphology. The PMMA-PS fibers obtained with ultrasound and magnetic assistance are finer, more uniform, and more smoothly surfaced, and they show a higher degree of alignment and ordering. Notably, numerous spindle-like structures (fusiform beads) appear along the fibers in the assisted process, whereas such structures were nearly absent in the conventional fibers. As shown in Figure 2c, the ultrasonically and magnetically assisted fibers have smaller average fiber diameters (~0.418 ± 0.13 μm for the thin fiber segments) and incorporate tightly packed spindle-shaped nodes (averaging ~3.22 μm in diameter) along their length. These spindle “knots” are well-integrated into the fiber network, appearing as elongated bead-like enlargements periodically distributed on the fibers. The overall fiber surface remains quite smooth and refined despite the presence of these spindle nodes, indicating that the process produces uniform core–shell structures rather than irregular beads.

**Figure 3:**
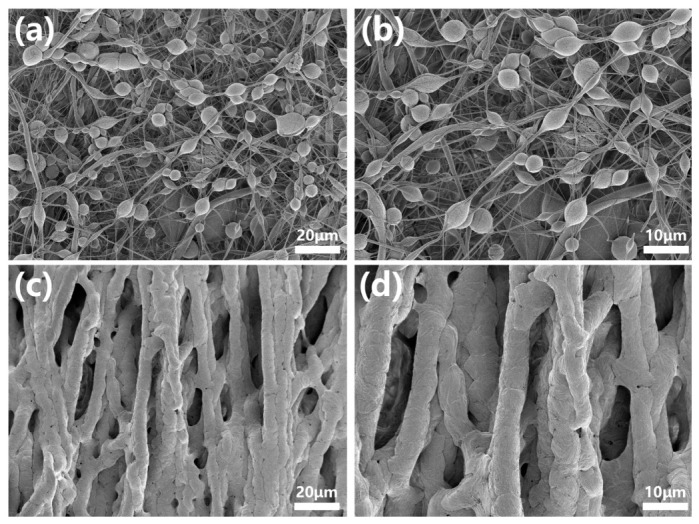
Multi-layered fibrous structure where upper fibers drape over lower fibers, creating a complex web of interconnected pores with significant overhangs. (**a**,**b**) SEM images of surface morphology for the conventional fibres with different magnifications. (**c**,**d**) SEM images of surface morphology for the 3D porous structure with different magnifications caused by the simultaneous ultrasonic and magnetic field.

The achievement of robust superhydrophobicity is therefore critically dependent on the surface topography. As theorized by Tuteja et al. and others, surfaces with ‘re-entrant’ or ‘doubly re-entrant’ curvature can support a stable composite interface even for liquids that would normally wet the material (i.e., θ < 90°). Our new high-magnification SEM images (Figure 3c,d) reveal that the electrospun membrane is not a simple 2D array but a complex 3D porous structure. At the micro-scale, the crossing and stacking of fibers (diameter ~500 nm) create a network of overhangs and concave geometries. At the nano-scale, the silica nanoparticles decorating the curved fiber surfaces introduce an additional level of re-entrant curvature. This hierarchical re-entrant structure presents a significant energy barrier that prevents the water droplet from sagging into the texture and displacing the trapped air, thereby stabilizing the Cassie-Baxter state against the Laplace pressure of the droplet.

The introduction of ultrasonic vibration and a magnetic field during electrospinning is responsible for these morphological differences. Ultrasonic agitation significantly reduces the spinning solution’s viscosity, which in turn promotes the formation of thinner fibers and spindle structures. Viscosity measurements show that the PMMA-PS solution’s viscosity drops from 7521 mPa·s (without ultrasound) to 5032 mPa·s after ultrasonic treatment. The ultrasound likely improves the dispersion of polymer molecules and disrupts their entanglements (by breaking and recombining molecular chains), effectively lowering the viscosity. A lower viscosity enables the solution to flow more easily and respond more dynamically to the electric field, increasing the vibration speed and amplitude of tiny fluid elements. Consequently, less force is needed to overcome viscous resistance, and more of the electrostatic drawing force can be applied to stretch and thin the fibers. Finer fibers facilitate more rapid solvent evaporation with minimal solvent residue on the fiber surface, yielding smoother fibers and greatly improving the solution’s spinnability. This improved spinnability is crucial for the formation of spindle-bead structures, as the jet thins and stretches more, the outer fluid can encapsulate the inner fluid intermittently to form the spindle knots.

In addition, the imposed magnetic field contributes to the morphological improvements by inducing better molecular orientation and jet stability. Under the magnetic field, the moving charged jet experiences a Lorentz force that helps stabilize its trajectory and encourages the polymer chains (once disentangled by ultrasound) to align in an orderly fashion. This results in fibers that are more uniformly drawn and deposited in an ordered arrangement. The combined effect of ultrasonic vibration and magnetic field thus produces a fibrous mat with highly ordered, refined fibers decorated with densely distributed spindle nodes (Figure 2d). Comparing Figure 2a,b, it is evident that the assisted process yields a much greater number of spindle structures that are packed more closely together. In summary, the ultrasound-magnetic coupling leads to a hierarchical fiber morphology (smooth, ultrafine fibers with periodic spindle-shaped enlargements) that was not attainable by conventional electrospinning alone.

### 3.2. Chemical Composition and Elemental Distribution

**Figure 4:**
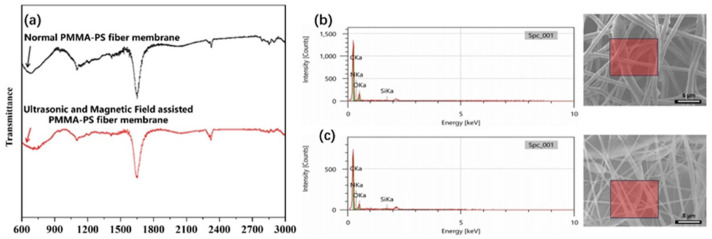
Chemical composition of PMMA–PS fibrous membranes. (**a**) FTIR spectra of membranes prepared with (black) and without (red) the coupled fields show identical signatures: benzene-ring vibrations (740–770 cm^−1^), C-O stretching (1390–1420 cm^−1^), C=O stretching (1690–1740 cm^−1^) and C=C- stretching (1630–1650 cm^−1^), confirming that both samples consist of the same PMMA/PS chemistry. (**b**,**c**) EDS profiles for the conventional and assisted fibres, respectively, reveal dominant C and O peaks (PMMA-PS) with only trace Si from the substrate, corroborating the FTIR results and indicating that the performance differences originate from morphology rather than composition.

Fourier-transform infrared (FTIR) spectroscopy was used to analyze the chemical composition of the electrospun fibers, and the spectra for fibers from both the conventional and the ultrasound/magnetic-assisted processes are presented in Figure 4a. Both samples exhibit identical characteristic absorption peaks, confirming that their chemical constituents are the same. Notably, strong absorption bands appear in the regions of 740–770 cm^−1^, 1390–1420 cm^−1^, 1630–1650 cm^−1^, and 1690–1740 cm^−1^ in both spectra. The peak at 740–770 cm^−1^ is indicative of aromatic ring vibrations, evidencing the presence of benzene rings from the PS component. The band at 1630–1650 cm^−1^ corresponds to C=C stretching (alkene bonds), further confirming the incorporation of polystyrene (which contains styrene double bonds in the aromatic ring). Meanwhile, the absorption in the 1390–1420 cm^−1^ range is assigned to C-O stretching, and the strong band at 1690–1740 cm^−1^ is characteristic of carbonyl (C=O) stretching; these features are signature peaks of the PMMA component (which has ester groups). Additionally, a smaller peak around 3024 cm^−1^ is observed, which can be attributed to residual N,N-dimethylformamide (DMF) solvent trapped in the fiber’s spindle structures. The FTIR results conclusively demonstrate that both PMMA and PS are present in the fibers produced by both methods. In other words, both the conventional electrospinning and the ultrasonic/magnetic coupled electrospinning yield composite fibers made of the same two polymers (PMMA and PS). There is no evidence of new chemical bonds or different functional groups emerging from the assisted process differences between the two fiber mats; therefore, they arise from physical morphology and structure rather than chemical composition.

Energy-dispersive X-ray spectroscopy (EDS) was employed to determine the elemental composition of the fibrous membranes. As shown in the EDS spectrum (Figure 4b,c), the PMMA-PS fiber mat produces intense signals for carbon (C) and oxygen (O), with C and O atomic percentages around 78.80% and 21.01%, respectively. This elemental composition is fully consistent with a PMMA/PS composite, since both polymers are composed chiefly of carbon (from the polymer backbones and aromatic rings) and oxygen (from the carbonyl and ester groups of PMMA). The presence of C=O and C-O bonds and benzene rings in the material (as confirmed by FTIR) aligns with the strong C and O peaks observed in EDS. A small amount of silicon (Si) signal is also detected, which is attributed to the underlying substrate (silicon wafer or glass) contributing a slight background signal due to electron beam penetration or signal reflection during analysis. In addition, a weak nitrogen (N) peak can be observed in the spectrum; this likely originates from residual DMF solvent (containing N atoms) or minor atmospheric adsorption on the fibers.

A comparison of the EDS results for the two fabrication methods (Figure 4b vs. Figure 4c) shows that both the conventional and the ultrasound/magnetic-assisted fiber mats have very similar elemental makeup. Each exhibits dominant C and O peaks (along with a minor N peak and a trace Si peak), and the relative ratios of these elements are essentially the same for both samples. While the absolute intensities of the peaks differ (owing to slight differences in sample thickness or beam interaction volume), the normalized composition is nearly identical. These EDS findings, together with the FTIR analysis, confirm that both types of fiber membranes are composed of the same PMMA and PS components. The enhanced performance observed in the ultrasonically and magnetically processed fibers is therefore not due to any compositional change, but purely a result of differences in microstructure and morphology imparted by the processing conditions.

### 3.3. Surface Roughness and Topography

**Figure 5:**
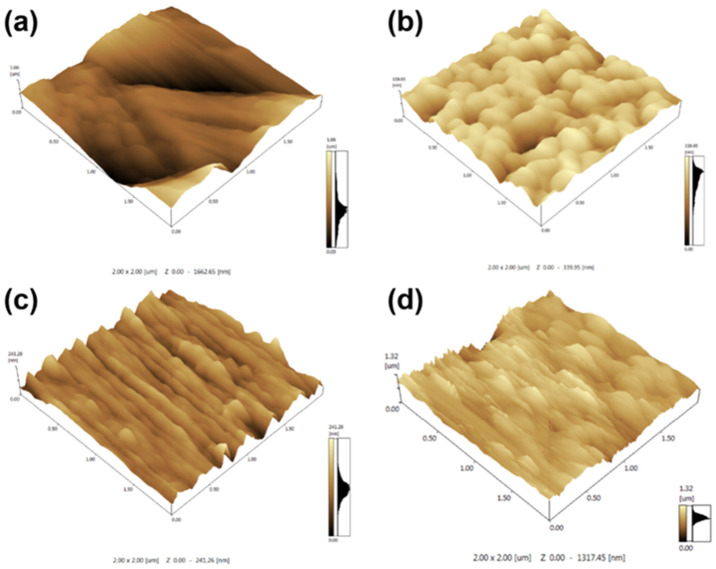
AFM three-dimensional topographies illustrating the evolution of surface roughness. (**a**) Conventional fibres form a relatively flat mat (R_a_ ≈ 1.16 µm). (**b**) Ultrasound-only processing introduces microscale protuberances (R_a_ ≈ 0.34 µm). (**c**) Magnetic-only processing generates regularly aligned ridges associated with spindle formation (R_a_ ≈ 0.26 µm). (**d**) Simultaneous ultrasound and magnetic assistance produces the roughest surface (R_a_ ≈ 1.32 µm) owing to uniform, large-amplitude spindle knots.

Atomic force microscopy (AFM) was used to characterize the surface topography and roughness of the fiber membranes. The 3D surface height maps in Figure 5 reveal significant differences in surface morphology among fibers produced under different conditions (traditional vs. ultrasound-assisted vs. magnetic-assisted vs. combined). In the conventionally electrospun PMMA-PS membrane (Figure 5a), the surface is relatively smooth and uniform. The fibers are tightly packed with minimal void space between them, resulting in a continuous mat with very few surface protrusions or depressions. The average surface height variation is around 1162 nm, which corresponds to the fiber diameter scale and confirms the SEM observation (Figure 2 and Figure 3) that the fibers are densely arranged and fairly even. The lack of pronounced peaks or gaps in the AFM image indicates that the traditional fibers form a flat, homogeneous surface with low roughness.

With the introduction of ultrasonic vibration during electrospinning (Figure 5b), the fiber mat’s surface becomes noticeably more textured. The AFM image of the ultrasound-assisted fibers shows an undulating surface with discernible peaks and valleys. Unlike the smooth surface of the control sample, this surface has a hierarchical structure: microscale protrusions are evident, and they are arranged in a somewhat periodic manner. The protrusions (burr-like features) are densely distributed, and clear gaps or valleys exist between fiber bundles. The average height of these surface asperities (peak-to-valley) reaches about 339 nm, indicating a moderate increase in surface roughness compared to the traditional sample. This increased roughness can be attributed to the more uneven fiber deposition and the beginning of spindle structure formation under ultrasonic assistance (even without a magnetic field).

When a magnetic field is applied during electrospinning (Figure 5c), the fiber surface morphology becomes even more structured. The magnetic-field-assisted PMMA-PS membrane shows a tendency toward more regular, aligned features on the nanoscale. The AFM image suggests that the PS and PMMA components have arranged such that PS envelops the PMMA fibers, forming the intended core–shell spindle architecture. As a result, the fibers display distinct spindle-shaped bulges (nodes) along their length, which translate into raised features on the surface. The presence of these spindle nodes causes the fiber mat to have more pronounced convexities and concavities on its surface. Compared to the traditional and even the ultrasound-only sample, the magnetic-assisted fibers have larger and more numerous surface protrusions. The “burrs” (peaks) and depressions in Figure 5c are more significant, reflecting the formation of the spindle knots, which increase the local fiber diameter at intervals. Overall, the surface is rougher than in the first two cases, although the arrangement of peaks and valleys is more regular due to the aligning influence of the magnetic field.

Finally, the combined ultrasonic and magnetic field assisted fibers exhibit the most extreme surface topology (Figure 5d). In this case, the spindle-bead structure of the fibers is highly developed. The spindle knots are very evident, relatively large, and uniformly distributed along the fibers. The AFM surface map reveals a dense array of peaks corresponding to these spindle knots, with intervening valleys where the thinner fiber segments lie. This sample shows the highest surface roughness among all the conditions examined. The protrusions and indentations on the surface are significantly taller and deeper, respectively, than those on the fibers produced by any other single-factor condition. The roughness is amplified by the regular spacing and sizable dimensions of the spindle structures. In essence, the simultaneous application of ultrasound and magnetic field yields a fiber membrane with a maximally rough, multi-scale surface, which, as will be discussed, is highly beneficial for achieving superhydrophobic behavior.

### 3.4. Enhanced Superhydrophobicity Performance

**Figure 6:**
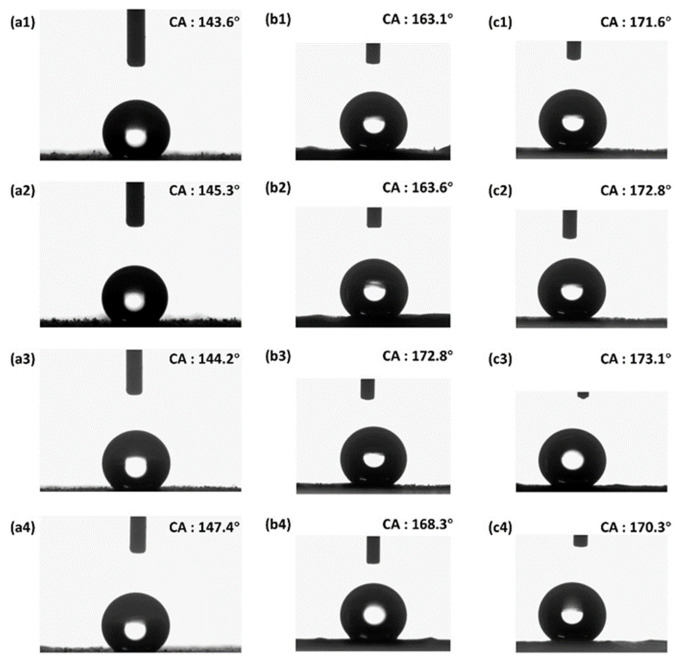
Static water-contact-angle (CA) images demonstrating the tunable wettability of PMMA–PS membranes. (**a1**–**a4**) Conventional fibres (four independent positions) display CAs of 143–147°, reflecting moderate hydrophobicity. (**b1**–**b4**) Ultrasound-only samples (15 wt % PMMA/PS) show enhanced CAs of 163–172°. (**c1**–**c4**) Ultrasound and magnetic-field-assisted fibres achieve superhydrophobicity, with CAs of 170–173° across multiple measurements, confirming the robustness of the coupled-field strategy.

As shown in Table 1, the wettability results demonstrate a dramatic improvement in water repellency for the fibers produced with the assisted coaxial process. The conventional electrospun PMMA-PS membrane (which already incorporates hydrophobic PS and SiO_2_ roughness) shows a water contact angle (WCA) of approximately 145°, indicating a hydrophobic surface. This value is in line with, though slightly higher than, typical electrospun PMMA-based membranes without special surface treatments (pure electrospun PMMA mats often have WCA in the 120–130° range). The enhanced WCA in our “conventional” sample can be attributed to the intrinsic hydrophobicity of PS and the modest roughness from the fibrous morphology and dispersed silica. However, it falls short of the 150° threshold for superhydrophobicity. In contrast, the membranes fabricated with ultrasonic vibration and magnetic field assistance exhibit significantly higher contact angles. In one implementation of the assisted process (Figure 6(b1–b4)), the WCA reaches around ~163–172°, and in the fully combined ultrasonic + magnetic field case (Figure 6(c1–c4)), the WCA advances to ~170–173°. These values place the surface squarely in the superhydrophobic regime (WCA > 150°). The increase of roughly 30° in WCA over the conventional fibers underscores the effectiveness of the dual-field-assisted coaxial electrospinning in tuning surface properties.

To understand the intrinsic wettability of our materials, the surface free energy (SFE) was determined for smooth, spin-coated films. The SFE was calculated to be 41.1 mN/m for PMMA, 40.7 mN/m for PS, and 40.9 mN/m for the PMMA/PS blend. The contact angle of water on these smooth surfaces was 68.2°, 88.1°, and 75.4°, respectively. These results confirm that the constituent materials are intrinsically hydrophilic or mildly hydrophobic, not low-energy materials.

The selection of 15% PMMA and 15% PS as the optimal concentration was based on a systematic evaluation of the resulting superhydrophobicity, as shown in Table 1. In electrospinning, fiber diameter generally increases with solution concentration due to higher viscosity, which hinders jet stretching [1]. Our goal was to maximize the hierarchical roughness created by the spindle-knot morphology, which requires a delicate balance. At lower concentrations (5% and 10%), the solution viscosity was insufficient to consistently form stable, spindle-decorated fibers. Conversely, at higher concentrations (20%), the increased viscosity began to suppress the instabilities needed for spindle formation, leading to smoother fibers and a slight decrease in the water contact angle (e.g., from 173.1° at 15% to 170.3° at 20% in group c). The 15% concentration combination provided the optimal solution rheology to generate a high density of well-defined spindle-knots, thereby maximizing surface roughness and achieving the peak contact angle of 173.1°.

As correctly pointed out, contact angle hysteresis is a critical parameter for defining superhydrophobicity. As shown in Table 1, the control sample exhibited a very high CAH of 45.3° ± 5.1°, indicating a high-adhesion ‘sticky’ Wenzel or metastable Cassie state. In striking contrast, the US + MF treated membrane displayed a CAH of 4.8° ± 1.2°. This value is well within the <10° (and often <5°) criterion for a true superhydrophobic, low-adhesion surface. This low hysteresis is direct evidence of a robust Cassie-Baxter state, where the droplet has minimal pinning sites and can roll off easily, which we confirmed by tilting the sample (>5° tilt angle).

It is worth comparing these results to other approaches in the literature. Coaxial electrospinning in previous studies, for example, achieved WCAs of about 158° by coating hydrophilic fibers with a fluoropolymer, and electrospun composite films of common polymers typically max out around 155–160° without fluorinated additives. Our strategy, despite using standard polymers (PMMA and PS) rather than specialized low-surface-energy fluoropolymers, exceeds these values; the ~173° contact angle observed is among the highest reported for electrospun fiber mats. Such a high static WCA suggests that water droplets rest on the assisted fiber membrane in a Cassie-Baxter state, supported by air pockets between the microscale fibers and the nanoscale. This conclusion is strongly supported by our sliding angle measurements, a critical parameter for evaluating water droplet mobility and self-cleaning potential [11]. As shown in Table 1, the water droplet on the conventional membrane (group a3) was strongly pinned (SA > 90°), indicative of a high-adhesion Wenzel or mixed wetting state. In contrast, the optimized superhydrophobic membrane (group c3) exhibited a very low sliding angle of only 4.2° ± 0.5°. This extremely low water adhesion confirms the presence of a stable Cassie-Baxter state, where the droplet sits atop trapped air pockets, allowing it to roll off with minimal tilting. This demonstrates the excellent self-cleaning potential of the membranes fabricated by our coupled-field method.

Indeed, qualitatively, droplets on the superhydrophobic membrane were almost perfectly spherical and rolled off with minimal tilt, indicating low contact angle hysteresis. This behavior is consistent with other superhydrophobic fiber systems, which exhibit roll-off (sliding) angles on the order of only a few degrees when the Cassie–Baxter condition is fully established, that is, when air pockets are stably trapped within the hierarchical surface texture and the solid–liquid contact fraction falls below a critical threshold. By contrast, water droplets on the conventional membrane tend to stick or slide only at much higher tilt angles, reflecting greater hysteresis likely due to more wetting of the fiber surface (Wenzel state wetting).

The novelty of the ultrasonic and magnetic field-assisted coaxial electrospinning method is evident in this context. The combination of ultrasonic dispersion of nanoparticles (ensuring each fiber’s surface is decorated with uniformly distributed nano-scale roughness) and magnetic-field-induced fiber orientation yields a multiscale textured, yet chemically homogeneous, surface. This one-step fabrication approach produces fibrous mats with superior water repellency without the need for any post-treatment (such as fluorination or plasma etching). The achieved superhydrophobic performance (contact angle ~173°) not only surpasses the conventionally electrospun counterpart by a wide margin but also outperforms many previously reported electrospun hydrophobic membranes. These results highlight the effectiveness of combining physical field aids with coaxial electrospinning to engineer surface structure. In summary, the assisted PMMA-PS/SiO_2_ membranes exhibit extreme water repellency (superhydrophobicity) and improved uniformity, demonstrating a clear enhancement in performance and functionality compared to conventionally electrospun membranes. This finding opens up possibilities for high-performance filtration, self-cleaning, or anti-fouling applications where such superhydrophobic fibrous surfaces are highly desirable.

The observation of a residual contact spot on the control and MF-only samples after the droplet was removed is indicative of high contact angle hysteresis and liquid pinning, characteristic of a ‘sticky’ Wenzel or metastable Cassie-Baxter state. In stark contrast, the US + MF sample exhibited very low contact angle hysteresis (<5°) and water droplets could roll off easily, confirming a low-adhesion, robust superhydrophobic surface.

Given that the smooth surfaces of our polymers are intrinsically hydrophilic (WCA < 90°), the observed superhydrophobicity on the electrospun membranes cannot be explained by the Wenzel model. Instead, it is attributed to the formation of a stable Cassie-Baxter wetting state. The dual-scale roughness—micrometer-sized fibers decorated with silica nanoparticles—creates a hierarchical topography that effectively sustains a composite solid-liquid-air interface. The high apparent contact angle is therefore a result of the water droplet resting predominantly on a cushion of trapped air.

### 3.5. Mechanism of the Superhydrophobic Effect

**Figure 7:**
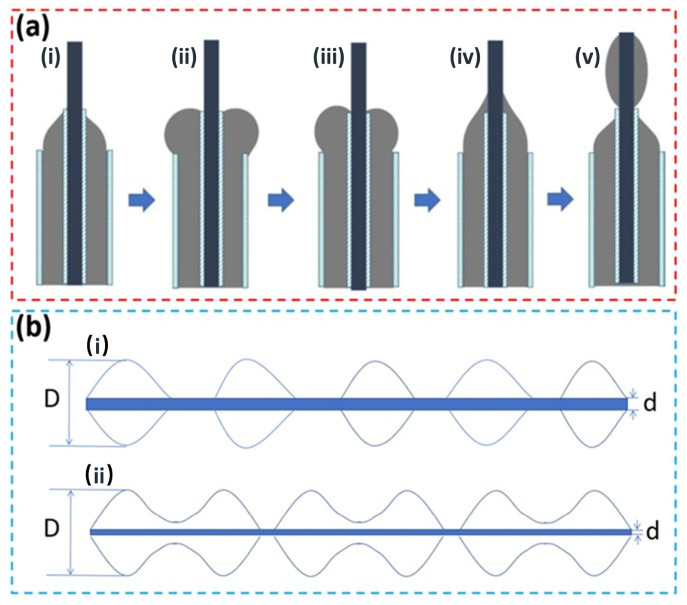
Proposed mechanism for spindle-knot generation and its role in surface roughness. (**a**) Sequential snapshots (i–v) of the coaxial jet illustrate bulging of the sheath solution, its encapsulation of the core, elongation under the electric field and solidification into a spindle knot. (**b**) Schematic comparison of diameter ratio A = D/d for conventional (i) and assisted (ii) fibres: ultrasound reduces the thin-segment diameter d whereas the knot diameter D is preserved, markedly increasing A and, hence, micro-/nano-scale roughness that drives the observed superhydrophobicity.

The superior superhydrophobicity observed in the ultrasonically and magnetically assisted fibers can be directly attributed to their unique spindle-beaded fiber morphology and the effects of the combined processing on fiber structure. A schematic illustration of the formation mechanism of the spindle-bead structure in coaxial electrospinning is provided in Figure 7. In a coaxial spinneret, two polymer solutions (here, a PMMA solution and a PS solution) are extruded concentrically, one forming the core and the other forming the shell of the compound jet. As the outer (sheath) solution is pumped out of the needle, it tends to bulge outward due to instabilities and surface tension differences, forming a droplet-like front (Figure 7a(i–iii)). When this bulging outer fluid advances enough to contact the inner core fluid (Figure 7a(iv)), the outer fluid envelops the inner fluid at that point. The electrostatic forces then draw this encapsulated portion forward, and as it stretches, the outer layer wraps around the inner core, creating a spindle-shaped composite droplet along the jet (Figure 7). This encapsulated droplet elongates as it travels and eventually solidifies into a spindle knot on the fiber, while the segment of the jet between two successive spindle knots forms the thinner spindle fiber region. In this way, the coaxial electrospinning process (especially under the right conditions) produces a fiber consisting of alternating thick and thin sections—the thick sections (spindle knots) result from periodic instabilities where the outer fluid collects around the inner fluid, and the thin sections are the continuous fiber that connects these knots. Each spindle unit is thus composed of a bulbous spindle knot and a slender fiber segment, and the spindle fiber segments typically have a uniform diameter and are closely packed in the fibrous mat, whereas the spindle knots protrude outward, creating surface asperities.

Under conventional processing conditions (without special assistance), the formation of such spindle structures is minimal or not pronounced, as observed in Section 3.1. However, when ultrasonic vibration and magnetic field are applied, the balance between the fiber thinning and bead formation shifts dramatically. An important metric to consider is the ratio of the spindle knot diameter (D) to the fiber segment diameter (d), denoted as A = D/d. This diameter ratio effectively correlates with the surface roughness introduced by the spindle structure: a larger spindle relative to the fiber thickness (higher A) creates a more pronounced topographical contrast on the fiber surface (greater roughness). In the ultrasound/magnetic-assisted fibers, the average fiber diameter *d* is significantly reduced (due to fiber refinement), while the spindle node diameter remains relatively large (comparable to the unassisted case or determined by the coaxial flow rates). Thus, the diameter ratio A is markedly increased for the assisted process. Thus, the diameter ratio A is markedly increased for the assisted process. We can quantitatively compare this value for the two methods. For the conventional fibers (Figure 2a), which lack spindle structures, D ≈ d ≈ 3.3 μm, yielding an A value of approximately 1. In stark contrast, for the ultrasound/magnetic-assisted fibers (Figure 2c), with a spindle diameter (D) of ~3.22 μm and a fiber segment diameter (d) of ~0.418 μm, the A value is calculated to be approximately 7.7. This nearly eightfold increase in the A ratio provides quantitative evidence for the dramatic enhancement in micro-scale surface roughness achieved with our novel method. Based on the quantitative analysis and the SEM images (Figure 2), it is clear that the coupled process yields much finer fibers (smaller *d*) yet still maintains or even increases the size and number of spindle knots (*D* and their frequency). Consequently, the surface roughness of the fibers (and of the fibrous membrane as a whole) is substantially enhanced in the presence of ultrasound and a magnetic field. Figure 7b schematically contrasts the surface structure of fibers from the two processes: the conventional fiber (Figure 7b(i)) has a relatively smooth surface with few protrusions, whereas the ultrasonically and magnetically processed fiber (Figure 7b(ii)) shows many spindle knots (protrusions) and deep recesses between them, corresponding to a highly rough surface at the micro-scale.

Several factors contribute to the increased spindle formation and roughness under the coupled field conditions. Ultrasonic vibration plays a critical role by reducing the solution viscosity and improving its flow characteristics, as discussed earlier. When the viscosity is lower, the polymer solution becomes more amenable to stretching and to the growth of instabilities. The reduced viscoelastic drag allows the charged jet to elongate more easily and can facilitate the periodic breakup or pulsation of the outer fluid around the inner fluid, which is necessary for spindle knot formation. In other words, a lower viscosity (achieved via ultrasound-induced disentanglement of polymer chains) increases the tendency for the outer solution to form droplets or bulges around the core, thereby promoting the generation of spindle beads. Furthermore, by continuously agitating the solution, ultrasound helps to prevent clogging of the coaxial needle and maintains a steady flow; this is important because any interruption or uneven flow can suppress the formation of regular spindle structures.

At the same time, the applied magnetic field exerts a stabilizing and ordering influence on the electrospinning jet. Through Lorentz forces acting on the moving charged jet, the magnetic field can constrain the jet’s erratic whipping motion, resulting in a more stable, straight jet trajectory towards the collector. This stability means that the spindle-forming instabilities, rather than being overwhelmed by chaotic whipping, can develop in a more controlled and periodic fashion. Additionally, the magnetic field encourages alignment of polymer molecular chains and the deposited fibers. Once the polymer chains have been partially disentangled by ultrasound, the magnetic field helps to orient them as the fiber solidifies, which can lead to a more uniform distribution of spindle nodes along the fiber. The magnetic force may also cause the deposited fibers to lie in a more aligned manner on the collector, contributing to the orderly arrangement observed in the assisted samples. In essence, the synergistic combination of ultrasound and magnetic field exploits the advantages of both: ultrasound ensures the solution is in an optimal state for forming fine fibers and beads (by lowering viscosity and preventing blockage), and the magnetic field ensures that these fibers and beads form in a regular, aligned manner (by stabilizing the jet and guiding fiber deposition). This effective coupling overcomes the individual limitations of each method (for instance, ultrasound-alone benefits can be transient as viscosity can creep back up when ultrasound is stopped, and magnetic-alone spinning might not achieve significant fiber thinning or smoothness by itself). The result is a process that consistently produces fibers with a much higher density of spindle structures and greater uniformity.

The dramatically increased surface roughness from the spindle structures is the key to the enhanced superhydrophobicity of the ultrasound/magnetic-assisted fiber mats. When the surface of a material has a high density of protrusions (spindle knots) and depressions, water droplets are less able to fully wet the surface, instead sitting on the tips of the microscale “roughness” features. In the case of the PMMA-PS fibers, the spindle knots act as tiny raised hills that support the water droplet, while air pockets remain trapped in the valleys (between spindle knots and between fibers). This composite solid-air surface beneath the droplet leads to a Cassie-Baxter state, where the droplet contacts a mixture of solid (polymer) at the spindle tips and air elsewhere. The Laplace pressure associated with the curvature of the droplet between the spindle knots further resists liquid penetration into the surface texture. In practical terms, the droplet is effectively suspended on the array of spindle knots, which significantly reduces the liquid–solid contact area and adhesion. As a result, the water droplet can easily roll off, and the apparent contact angle is very high (as observed, >170°). Importantly, since the chemical composition of both the traditional and assisted fibers is the same (both are PMMA-PS composites), the difference in wettability can be directly attributed to the difference in surface morphology. When the intrinsic material surface energies are similar, increasing the surface roughness is the dominant factor in boosting hydrophobicity. The coupled ultrasonic and magnetic electrospinning technique maximizes this roughness by generating an abundance of spindle microstructures, thereby achieving the best superhydrophobic performance.

In summary, the ultrasonic vibration and magnetic field coupling method provides a powerful means to tailor the microstructure of coaxially electrospun fibers, leading to significant enhancements in surface properties like wettability. The mechanism involves ultrasound-induced viscosity reduction (improving fiber fineness and spindle formation) and magnetic-field-induced jet stabilization and molecular alignment (improving fiber ordering and uniformity of spindle distribution). Together, these effects result in fibers with smaller diameters, more numerous spindle-bead protrusions, and highly ordered arrangements, which translate into a much rougher surface texture. The rough, spindle-decorated fibers, in turn, create the necessary topography for extreme water repellency. Additionally, from a processing perspective, the combined use of ultrasound and a magnetic field addresses practical challenges: it mitigates needle clogging (by keeping the solution less viscous and continuously agitated), counteracts the tendency of the solution’s viscosity to increase after prolonged spinning, and improves fiber uniformity and smoothness beyond what a magnetic field alone could achieve. Therefore, the coupling of ultrasonic vibration with a magnetic field not only yields superhydrophobic PMMA-PS fiber mats with optimal surface roughness but also enhances the reliability and efficiency of the electrospinning process itself. The outcomes of this study suggest that such a coupled-field electrospinning approach could be broadly beneficial for fabricating functional fibrous materials where controlled morphology and surface functionality are desired.

## 4. Conclusions

In this study, we developed a simple and efficient method to produce PMMA-PS fiber membranes with outstanding superhydrophobic properties using ultrasound and magnetic-field-assisted coaxial electrospinning. This combined technique significantly improves fiber structure, creating uniform, spindle-shaped features that greatly enhance surface roughness. Chemical analyses confirmed that the enhanced superhydrophobicity arises from changes in surface structure rather than chemical composition. The optimized membranes achieved water contact angles as high as 173.1°, substantially better than conventionally prepared fibers (~143.6°). Our results highlight that ultrasound and magnetic fields can effectively control fiber morphology and wettability. This one-step, field-assisted electrospinning approach enables scalable fabrication of superhydrophobic PMMA-PS films without fluorine doping or post-treatment, opening new avenues for cost-effective anti-wetting materials in environmental remediation and smart textiles. Future studies can further explore material combinations and refine process parameters to expand practical applications.

## Figures and Tables

**Table 1:** Statistical records of superhydrophobic behaviors on PMMA and PS composite fibers with different concentrations.

Group	PMMA + PS Concentrations	WCA Average Data	Sliding Angle	CAH Data
a1	5% + 5%	143.6° ± 0.2°	4.2° ± 0.6°	23.2° ± 0.3°
a2	10% + 10%	145.3° ± 0.3°	5.3° ± 0.5°	36.4° ± 1.7°
a3	15% + 15%	144.2° ± 0.2°	3.7° ± 0.4°	45.3° ± 5.1°
a4	20% + 20%	147.4° ± 0.1°	3.2° ± 0.3°	24.9° ± 1.1°
b1	5% + 5%	163.1° ± 0.3°	5.4° ± 0.4°	30.5° ± 1.6°
b2	10% + 10%	163.6° ± 0.2°	6.1° ± 0.5°	27.3° ± 0.6°
b3	15% + 15%	172.8° ± 0.3°	6.5° ± 0.7°	10.6° ± 0.4°
b4	20% + 20%	168.3° ± 0.1°	5.5° ± 0.4°	12.7° ± 1.1°
c1	5% + 5%	171.6° ± 0.3°	6.2° ± 0.6°	8.3° ± 1.4°
c2	10% + 10%	172.8° ± 0.2°	3.6° ± 0.3°	6.7° ± 0.9°
c3	15% + 15%	173.1° ± 0.1°	4.2° ± 0.5°	4.8° ± 1.2°
c4	20% + 20%	170.3° ± 0.2°	5.7° ± 0.6°	5.1° ± 0.7°

## Data Availability

The original contributions presented in this study are included in the article. Further inquiries can be directed to the corresponding author.

## References

[1] Hong W., Woo H.J., Choi H.W., Kim Y.S., Kim G.D. (2001). Optical property modification of PMMA by ion-beam implantation. Appl. Surf. Sci..

[2] Zhang R., Ma Y., Lan W., Sameen D.E., Ahmed S., Dai J., Qin W., Li S., Liu Y. (2021). Enhanced photocatalytic degradation of organic dyes by ultrasonic-assisted electrospray TiO_2_/graphene oxide on polyacrylonitrile/β-cyclodextrin nanofibrous membranes. Ultrason. Sonochem..

[3] Abdelsayed V., Alsharaeh E., El-Shall M.S. (2006). Catalyzed radical polymerization of styrene vapor on nanoparticle surfaces and the incorporation of metal and metal oxide nanoparticles within polystyrene polymers. J. Phys. Chem. B.

[4] Matusinović Z., Rogošić M., Šipušić J. (2009). Synthesis and characterization of poly(styrene-co-methyl methacrylate)/layered double hydroxide nanocomposites via in situ polymerization. Polym. Degrad. Stab..

[5] Coşkun M., Seven P. (2011). Synthesis, characterization and investigation of dielectric properties of two-armed graft copolymers prepared with methyl methacrylate and styrene onto PVC using atom transfer radical polymerization. React. Funct. Polym..

[6] Rozik N.N., Khalaf A.I., Ward A.A. (2016). Studies the behaviors of polyaniline on the properties of PS/PMMA blends. Proc. Inst. Mech. Eng. Part L J. Mater. Des. Appl..

[7] Alsaad A., Al-Bataineh Q.M., Ahmad A., Jum’h I., Alaqtash N., Bani-Salameh A. (2020). Optical properties of transparent PMMA-PS/ZnO NPs polymeric nanocomposite films: UV-Shielding applications. Mater. Res. Express.

[8] Zeng X.-F., Kong X.-R., Ge J.-L., Liu H.-T., Gao C., Shen Z.-G., Chen J.-F. (2011). An effective solution mixing method to fabricate highly transparent and optical functional organic−Inorganic nanocomposite film. Ind. Eng. Chem. Res..

[9] Sharma K., Dixit M. (2010). Mechanical and Thermal Transport Properties of PMMA/PC and PMMA/PS Blends. Proceedings of the 5th National Conference on Thermophysical Properties: (NCTP-09), Baroda, India, 7–9 October 2009.

[10] Das A., Dey A.B., Chattopadhyay S., De G., Sanyal M.K., Mukherjee R. (2020). Nanoparticle induced morphology modulation in spin coated PS/PMMA blend thin films. Langmuir.

[11] Sriboonruang A., Kumpika T., Sroila W., Kantarak E., Singjai P., Thongsuwan W. (2018). Superhydrophobicity/superhydrophilicity transformation of transparent PS-PMMASiO_2_ nanocomposite films. Ukr. J. Phys..

[12] Ma Y., Cao X., Feng X., Ma Y., Zou H. (2007). Fabrication of super-hydrophobic film from PMMA with intrinsic water contact angle below 90°. Polymer.

[13] Turkoglu Sasmazel H., Alazzawi M., Kadim Abid Alsahib N. (2021). Atmospheric pressure plasma surface treatment of polymers and influence on cell cultivation. Molecules.

[14] Hoang C.H., Nguyen T.T., Ho D.Q., Le H.V., Nguyen H.H. (2023). Fabrication of superhydrophobic surfaces for applications in total internal reflection effects. Mater. Today Commun..

[15] Stacey S., Narasimha P., Christopher C., Lisa K., Bradley A., Brian C., Fow-Sen C., Singh N.B. (2019). Importance of lotus effect on surface sensing. Proc. SPIE.

[16] Gao J., Huang X., Wang L., Zheng N., Li W., Xue H., Li R.K., Mai Y.-W. (2017). Super-hydrophobic coatings based on non-solvent induced phase separation during electro-spraying. J. Colloid Interface Sci..

[17] Lalire T., Otazaghine B., Longuet C., Taguet A. Control of graphene localization in co-continuous PMMA/PS polymer blends via chemical modification for electrical application. Proceedings of the C’Nano 2020—The Nanoscience Meeting.

[18] Cai H., Zheng B., Zhu D., Wu Y., Cardinaels R., Moldenaers P., Shen Z., Sheng Y., Zhu H., Yu K. (2023). Regulation of dewetting and morphology evolution in spin-coated PS/PMMA blend films via graphene-based Janus nanosheets. Appl. Surf. Sci..

[19] Watanabe S., Fujisaki M., Murai K., Matsumoto M. (2018). Superhydrophobic Surfaces on Phase-separated Nanostructures of Polystyrene/Polymethyl Methacrylate Films Fabricated by the Double-spray Technique. J. Oleo Sci..

[20] Bürger J., Kunnathully V.S., Kool D., Lindner J.K.N., Brassat K. (2020). Characterisation of the PS-PMMA Interfaces in Microphase Separated Block Copolymer Thin Films by Analytical (S)TEM. Nanomater.

[21] Rahmani S., Arefazar A., Latifi M. (2017). PMMA/PS coaxial electrospinning: A statistical analysis on processing parameters. Mater. Res. Express.

[22] Xue J., Wu T., Dai Y., Xia Y. (2019). Electrospinning and Electrospun Nanofibers: Methods, Materials, and Applications. Chem. Rev..

[23] An A.K., Guo J., Lee E.-J., Jeong S., Zhao Y., Wang Z., Leiknes T. (2017). PDMS/PVDF hybrid electrospun membrane with superhydrophobic property and drop impact dynamics for dyeing wastewater treatment using membrane distillation. J. Membr. Sci..

[24] Rahmani S., Arefazar A., Latifi M. (2017). PMMA/PS coaxial electrospinning: Core–shell fiber morphology as a function of material parameters. Mater. Res. Express.

[25] Serrano-Garcia W., Ramakrishna S., Thomas S.W. (2022). Electrospinning Technique for Fabrication of Coaxial Nanofibers of Semiconductive Polymers. Polymers.

[26] Satheesh A., Alagiriswamy A., Devanand S., Nithiyanantham S. (2020). Solvent Assisted Coaxial-Electrospun Poly Methyl Methacrylate Polymer and Study of Resultant Fibers. Sens. Lett..

[27] Estrada-Monje A., Zitzumbo-Guzmán R., Bañuelos-Díaz J.A., Zaragoza-Contreras E.A. (2019). Ultrasonic dispersion and activation of TiO_2_ nanoparticles and its effect on bacterial inhibition in EVA films. Mater. Chem. Phys..

[28] Baig M.I., Ingole P.G., Jeon J.-d., Hong S.U., Choi W.K., Lee H.K. (2019). Water vapor transport properties of interfacially polymerized thin film nanocomposite membranes modified with graphene oxide and GO-TiO_2_ nanofillers. Chem. Eng. J..

[29] Cao Q., Wan Y., Qiang J., Yang R., Fu J., Wang H., Gao W., Ko F. (2014). Effect of sonication treatment on electrospinnability of high-viscosity PAN solution and mechanical performance of microfiber mat. Iran. Polym. J..

[30] Si N., Xu L., Wang M.Z., Liu F.J. (2014). Effect of Ultrasonic Vibration on Electrospun Poly(vinyl Alcohol) (PVA) Nanofibers. Adv. Mater. Res..

[31] Qiang J., Wan Y.Q., Yang L.N., Cao Q.Q. (2013). Effect of Ultrasonic Vibration on Structure and Performance of Electrospun PAN Fibrous Membrane. J. Nano Res..

